# Folding *versus* aggregation: Polypeptide conformations on competing pathways

**DOI:** 10.1016/j.abb.2007.05.015

**Published:** 2008-01-01

**Authors:** Thomas R. Jahn, Sheena E. Radford

**Affiliations:** Astbury Centre for Structural and Molecular Biology, Institute of Molecular and Cellular Biology, University of Leeds, Mount Preston Street, Leeds LS2 9JT, UK

**Keywords:** Energy landscape, Protein folding, Protein misfolding, Aggregation, Amyloid fibril formation, Intermediate states, Oligomers

## Abstract

Protein aggregation has now become recognised as an important and generic aspect of protein energy landscapes. Since the discovery that numerous human diseases are caused by protein aggregation, the biophysical characterisation of misfolded states and their aggregation mechanisms has received increased attention. Utilising experimental techniques and computational approaches established for the analysis of protein folding reactions has ensured rapid advances in the study of pathways leading to amyloid fibrils and amyloid-related aggregates. Here we describe recent experimental and theoretical advances in the elucidation of the conformational properties of dynamic, heterogeneous and/or insoluble protein ensembles populated on complex, multidimensional protein energy landscapes. We discuss current understanding of aggregation mechanisms in this context and describe how the synergy between biochemical, biophysical and cell-biological experiments are beginning to provide detailed insights into the partitioning of non-native species between protein folding and aggregation pathways.

Despite their molecular diversity, proteins possess the common property that, in general, they can rapidly and spontaneously self-assemble into elaborate three-dimensional structures that are required for their specific functions. How this molecular feat is achieved is a fascinating, but still unanswered, question. Since the discovery that the native conformation is not the only fold available to a polypeptide chain, but an alternative structure, known as amyloid, may represent the primordial ground state of protein folding and assembly reactions, research into the specific features of polypeptides that ensure folding to the correct, functional native state and how off-pathway species are avoided, has increased in momentum enormously. Most importantly, the synergy between theoretical studies, biophysical experiments, cell biology and medicine is now beginning to provide common principles that rationalise the sequence-specific partitioning of polypeptides between protein folding and aggregation, as well as its pathological consequences.

This review describes the current molecular understanding of the mechanisms of protein folding and protein aggregation. We start by discussing current concepts of protein ensembles, derived from a combination of experiment, theory and simulation, in terms of kinetically meta-stable states on the energy landscape. The properties of intermediate states are particularly highlighted, as these species have been shown to be of particular importance in determining the diversion of molecules between the folding and aggregation landscapes. We then describe recent advances in theoretical frameworks, as well as the development of experimental techniques that, together, are beginning to reveal the structural complexity of states populated at different locations on the energy landscape, as well as the kinetic pathways that link them. Subsequently, the intrinsic properties of the polypeptide chain that shape the energy landscape in favour of functional folded proteins and the influence of extrinsic, cellular factors in shaping the landscape will be discussed, highlighting in particular how evolution has solved the ‘protein folding problem’ by establishing amino acid sequences that fold into stable proteins with desired functions whilst avoiding non-native, non-functional conformers. Finally, we conclude by pointing out the challenges that lie ahead in our quest to understand protein folding energy landscapes in atomistic detail and how we might hope to utilise this knowledge to control the partitioning between folding and aggregation in the future.

## Towards the description of protein conformational ensembles

### The folding side of the protein energy landscape

**Fig. 1:**
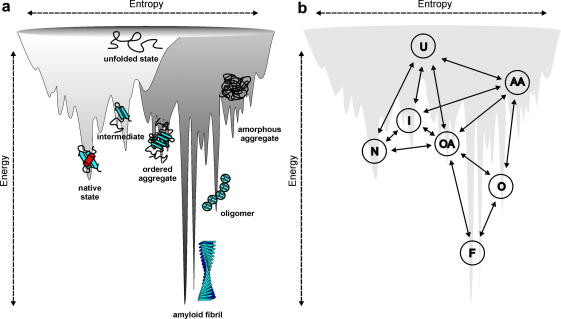
Illustration of a combined energy landscape for protein folding and aggregation. (a) The surface illustrates the roughness of the protein energy landscape, showing the multitude of conformational states available to a polypeptide chain. While rather simple folding funnels (light grey) can describe the conformational search of a single polypeptide chain to a functional monomer, intermolecular protein association dramatically increases ruggedness (dark grey). (b) Proposed pathways linking the conformational states shown in (a) populated on the combined folding and aggregation energy landscape.

The fact that protein folding must occur within a biologically feasible timescale excludes the possibility of a random-search mechanism for protein folding [1]. Therefore, and as a general principle, kinetic pathways lay the foundation to an ordered protein folding reaction. Although the complete folding trajectory of only very simple proteins has been determined at atomic-level resolution to date [2], biophysical and theoretical experiments picture protein folding, in general, as a sequential and highly cooperative reaction [3]. To envision the conformational space available to each individual polypeptide sequence under a given condition, the concept of energy landscapes was introduced [4,5]. This theoretical formalism describes the progression of unfolded polypeptide chains along an energy landscape towards the compact native structure (Fig. 1). For small proteins this landscape appears to be funnel-like and represents the evolutionary selection of polypeptide sequences able to fold rapidly and reliably towards a unique native state [6]. On the other hand, larger polypeptide sequences have rougher energy landscapes, allowing the population of partially folded species that may be on- or off-pathway to the native fold [7,8]. Characterising the multitude of conformational states populated on this energy landscape is not only crucial for developing an understanding of the determinants of protein folding and aspects of protein function, but it also contains crucial clues about side-reactions such as protein aggregation [9,10]. Such a characterisation involves describing the structural, kinetic and thermodynamic properties of all conformations accessible to a given polypeptide sequence. Obtaining this description has proved to be immensely challenging, however, as techniques able to resolve and structurally characterise these rapidly interconverting species needed to be developed, as well as the theoretical framework to interpret them. Nonetheless, using an array of ingenious experiments and by combining these with simulations and new theories, detailed insights into at least the most highly populated conformational ensembles on the protein folding landscape and the transition state barriers that separate them have emerged over the recent years.

### Unfolded states

Over the last decade enormous progress has been made in describing the conformation of unfolded states. These important species not only define the starting point for protein folding reactions [11], but for natively unfolded chains, may have important roles in a variety of biological processes [12]. While proteins unfolded with high concentrations of chaotropes or at high temperature initially led to the impression that unfolded states are random coils that lack persistent non-random inter-residue interactions [13], this view has changed over recent years as a result of an increasing number of detailed studies on denatured states under a range of solution conditions, principally using NMR^1^ spectroscopy [10]. Current views consider that, even in high concentrations of denaturant, significant clustering of aliphatic and aromatic side chains may persist, even if no significant secondary structure is retained [14,15]. Under less harsh conditions, in which some proteins can be tricked into being unfolded in the absence of denaturant (for example by mutating the sequence or changing the pH), the unfolded state has been shown to contain significant native-like interactions formed in concert with substantial numbers of both native and non-native side chain contacts [16,17]. In the case of the drk-SH3 domain a direct characterisation of the unfolded state has been possible due to the fact that the unfolded state is populated at equilibrium with the native protein under non-denaturing conditions. Using circular dichroism (CD), fluorescence and NMR spectroscopy, Crowhurst et al. were able to provide evidence for cooperative interactions in this unfolded state, based on non-native hydrophobic clusters around tryptophan residues [18]. Importantly, non-native interactions involving aromatic clusters have also been shown to persist in high denaturant concentrations in the case of lysozyme [14]. Fersht and coworkers have recently used a series of high-resolution NMR experiments to picture the structure of the denatured state of the *Drosophila* Engrailed Homeodomain (En-HD). Using the mutant protein L16A, a variant that is unfolded under conditions in which the wild-type sequence is native, the unfolded state was shown to be highly structured, possessing substantial amounts of native secondary and tertiary structure [19]. This denatured state under physiological conditions is reminiscent of protein folding intermediates found in other proteins [20–22] and highlights the difficulty in defining species as physiologically unfolded or intermediate when populated very early in folding [23]. Single molecule spectroscopy, in particular Förster resonance energy transfer (FRET), has been extremely powerful in separating heterogeneous subpopulations [24], thereby allowing the analysis of unfolded species even when present as minor conformations with the native protein [25,26]. This approach, in combination with CD and small angle X-ray scattering (SAXS) measurements, has indicated a dramatic collapse of unfolded structures upon removal of denaturant and suggests that secondary structure, as well as long-range inter-residue interactions, may form in the unfolded ensembles that initiate the folding reaction [27,28].

Residual structure in the unfolded ensemble has not only been suggested to reduce the conformational search during folding, but for some proteins may also play a role in the onset of protein aggregation of unfolded polypeptides [29,30]. NMR studies of the denatured states of amyloidogenic proteins have been performed for several proteins, including lysozyme [31], β_2_-microglobulin (β_2_m) [32] and an SH3 domain [33]. Although the acid-denatured states of these proteins are substantially disordered and dynamic, ^15^N relaxation experiments indicate that these ensembles contain substantial non-native short-range and long-range interactions, particularly involving the clustering of aliphatic and aromatic side chains, resulting in partially restricted conformations containing clusters of hydrophobic residues [31–33]. Although the effect of these hydrophobic clusters on amyloid formation is not fully understood, changes in the residual structure of the unfolded state can result in modulation of fibril formation rates [30], an observation that has also been found in protein folding studies [34]. Numerous experimental techniques [35], as well as theoretical studies [36], have indicated that a significant fraction of the contacts that define the native structure of a protein may already be present in the unfolded state, and their presence may be required to establish the overall native topology required for an effective folding reaction [37]. Interestingly, these structural preferences, determined by the intrinsic properties of the amino acid sequence, e.g. secondary structure propensity, charge and hydrophobicity, not only determine the ability of the polypeptide chain to fold efficiently to the native structure, but seem to be equally important in determining protein aggregation kinetics (see below). Therefore, Nature has had a difficult task in evolving sequences able to fold to a functional structure whilst avoiding off-pathway reactions that may lead to deadly oligomeric and/or polymeric structures.

### Intermediates and transition state ensembles (TSE)

The role of intermediates (i.e. populated partially folded states) in protein folding has been a long-standing question [8]. Based initially on the observation that many small proteins fold with apparent two-state kinetics [38], folding intermediates were initially thought to be aberrant misfolds on the folding energy landscape that represent off-pathway species or kinetic traps [39]. Today the increase in the power of experimental methods has resulted in the number of proteins folding *via* a pure two-state mechanism declining to only one or two remaining examples [8]. For example, experiments capable of measuring folding events on a μs timescale (e.g. using ultra-rapid mixing [40]), or of detecting rare species (e.g. using FRET, fluorescence correlation spectroscopy (FCS) or NMR spectroscopy [24,41]) have revealed partially folded states populated during the folding of even the simplest proteins [42,43]. In particular NMR relaxation dispersion measurements are highly sensitive for the detection of minor populations (down to 1%) of non-native protein conformations and can provide residue specific information on the kinetic and thermodynamic properties of these species [44,45]. Molecular dynamics (MD) simulations have also predicted the presence of intermediates in proteins initially characterised as two-state, guiding subsequent protein engineering experiments to stabilise such species so that they can be detected experimentally [46]. The length of polypeptide chain needed to span the structural and functional universe of proteins results in most proteins being over 200 residues in length. Such proteins usually possess multiple domains and, as a consequence, result in rough energy landscapes [8,9]. Larger chains have a higher tendency to collapse in aqueous solution, resulting in the formation of compact states which can contain substantial elements of native-like structure [47,48]. Reorganisation of inter-residue contacts (including both native and non-native interactions) in these compact states may involve high energy barriers, leading to the transient population of partially folded intermediate states [7]. Such species can be productive for folding (on-pathway) [49,50] or trapped such that the native structure cannot be reached without substantial reorganisational events (the intermediate is off-pathway) [51,52]. While a decade ago, the presence of folding intermediates was mainly inferred from the non-linear dependence of the folding kinetics (analysed on a log timescale) on the concentration of denaturant, or a burst phase (apparent missing amplitude) in the initial spectroscopic signal, technical and methodical advances now allow the formation of intermediates to be directly monitored (Table 1). Using these and other techniques, early kinetic intermediates have been shown to resemble the molten-globule intermediates found many years ago for several proteins under mildly denaturing conditions [53], suggesting that these equilibrium states can be considered as stable models of transient intermediates. Baldwin, Wright and colleagues have extensively studied the relationship between the equilibrium and kinetic folding intermediates of apomyoglobin. Using CD, fluorescence and NMR spectroscopy, together with quenched-flow hydrogen exchange (HX) and stopped-flow experiments, the kinetic and equilibrium intermediates of apomyoglobin have been shown to possess very similar conformational properties [21,54,55]. Similar results have achieved previously and since for RNase H [56], T4 lysozyme [57] and Im7 [58]. Trapping transiently populated species at equilibrium allows their analysis using a variety of structural techniques (Table 1) and thus offers a powerful and general method of determining the structures of non-native states on the folding energy landscape.

All species on the protein energy landscape are heterogeneous and dynamic, with species most distant from the native structure possessing the least ordered and thereby the most highly dynamic conformations. Rather than being able to define the structures of non-native states, therefore, as can be performed classically for native proteins using NMR or X-ray methods, non-native species are better described as conformational ensembles. Phi-value analysis, a pioneering protein engineering technique introduced by Fersht and coworkers [59], has been used extensively to picture the ensemble of conformations that make up the transition state ensemble (TSE), whose formation is the rate-limiting event in protein folding reactions. As long as appropriate and carefully designed mutations are made [60], phi-value analysis can reveal the role of individual side chain moieties in stabilising the TSE and this technique has now been used to determine the structural properties of a large number of proteins [61]. The results revealed a surprising simplicity to the folding process, where the sequence of events is determined predominantly by the topology of the native state [62]. The very rapid and efficient search to the native state is encoded by a network of interactions between key residues, forming a folding nucleus that establishes the native topology in the TSE [37]. In the case of the 98-residue protein acylphosphatase (AcP), as few as three residues are sufficient to determine an efficient search to the native topology of this α/β protein [63]. Studies on the ribosomal protein S6 have indicated the existence of specific, competing nuclei for a given protein fold, resulting in shifting dominant folding channels upon sequence divergence [64]. Furthermore, using a statistical coupling approach which takes the cooperative nature of amino acid interactions into account, Ranganathan and colleagues were able to show the importance of the specific distribution of just a few conserved protein interactions in determining a protein fold [65], opening the door to the design of new sequences capable of efficient folding to a functional state.

**Fig. 2:**
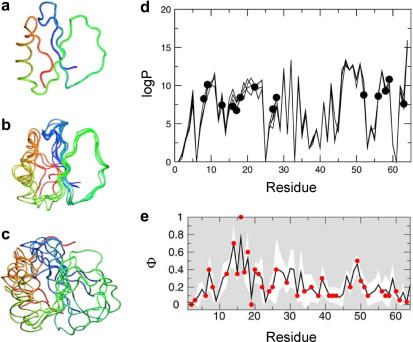
Application of restrained MD simulations to describe the conformational properties of dynamic ensembles. Structural ensembles of CI2 corresponding to (a) the crystal structure of the native state, (b) the native state ensemble determined from hydrogen exchange data and (c) the TSE determined using experimental phi-values. (d) Comparison of experimental protection factors (black circles) with those back-calculated (solid lines) from the native state ensemble shown in (b). (e) Agreement between the phi-values calculated from the TSE ensemble shown in c (black line) and experimental phi-values (red circles). Figure adapted from [71] with permission.

In the case of populated intermediates, experimental measures such as phi-values, hydrogen exchange protection factors or chemical shifts can be used to explore their conformational properties [66–68]. However, a direct interpretation of these measurements in structural terms is problematic since often the information is limited relative to the all-atom description required for a full understanding of their structural properties. In addition, the identity of the contacts made that give rise to the experimental measure is often not known. To maximise the information content of these experiments, therefore, new methods are currently being developed that combine MD simulations with experimental measurements to reveal atomistic models of these structural ensembles [10]. Using this approach, Vendruscolo and coworkers have revealed new insights into structural properties of the unfolded state of Δ131Δ [69], the intermediate state of the bacterial immunity protein Im7 [70], the rate determining transition state of AcP [63] and the native state of chymotrypsin inhibitor 2 (CI2) [71]. Fig. 2 shows the application of MD simulations restrained using hydrogen exchange protection factors to define the conformational properties of the native state ensemble of CI2, as well as phi-values to define the TSE. The results highlight the large equilibrium fluctuations encountered by the native structure, as well as the overall native topology of the folding transition state [71]. Importantly, double mutant cycles have been used to test the predictions of the TSE structure determined for barnase using phi-values to restrain MD simulations [72], whilst in the case of Im7, cross-validation between the ensembles calculated using different experimental parameters (hydrogen exchange protection factors, NMR chemical shifts and phi-value measurements) as restraints [70], has also validated the structures of its folding intermediate using this approach. Advancing techniques such as FRET-measurements or NMR-derived parameters (e.g. residual dipolar couplings, paramagnetic relaxation enhancement, or relaxation dispersion (see Table 1)), are beginning to provide routes capable of describing complex and heterogeneous populations, and even oligomeric structures, in unprecedented detail [73], providing further experimental data that can be used to test and refine the restrained MD procedures.

### Native states

Even under highly native conditions, structured proteins have access to a manifold of near-native conformations. It has recently become increasingly evident that the native structures of proteins show fluctuations around the minimal energy conformation that may result in the X-ray structure [74]. Such movements are required in order to encompass function, such as enzyme catalysis or ligand binding, as well as site-to-site communication within the globular protein fold or between protein subunits [75]. Wright and colleagues recently described the coupling between functional low-energy states populated sequentially in the catalytic cycle of dihydrofolate reductase (DHFR) in exceptional detail using a series of high-resolution NMR experiments [76]. The results indicated the existence of a series of ‘precast’ low-energy states, made sequentially available due to specific ligand binding that preexist within the native ensemble, that play a key role in enzyme catalysis. Similar experiments have revealed discrete conformational species within the native ensemble of signaling molecules, allosteric enzymes and membrane proteins [41,77,78]. Native state hydrogen exchange has provided particularly valuable insights into the stability of individual residues in native proteins, showing clear deviations from ‘all-or-nothing’ protein unfolding dynamics and revealing rarely populated non-native structures accessible by conformational fluctuations from the native state [68]. For example, Bai and colleagues have used measurements of the denaturant-dependence of hydrogen exchange rates to show that proteins undergo multiple localised unfolding reactions in all regions of the protein structure [79]. These fluctuations give rise to ‘partially unfolded forms’ (PUFs) which have been shown to exist even for the smallest protein domains found to date [80]. These ‘hidden intermediates’ cannot be observed using common spectroscopic techniques in bulk kinetic experiments, as they are usually populated after the rate-limiting transition state on the folding trajectory. In the case of apoflavodoxin, a series of studies by Bollen et al. have indicated the existence of kinetic and equilibrium folding intermediates [81]. Importantly, several of the PUFs are energetically close to the native state, but off the folding pathway. Whether these PUFs can be stabilised by the binding of ligands (and therefore may represent functional species) or whether their population increases due to point mutations, giving rise to the population of aggregation-competent states, remains to be shown. In the case of lysozyme, hydrogen exchange experiments monitored using mass spectrometry have revealed the link between dynamics in the native state and amyloid fibril formation [82]. While the point mutants I56T and D67H do not alter the structure of the native fold, they increase the rate of local unfolding events, resulting in an increase in the population of the aggregation precursor species. Similarly, isomerisation of a single *cis*-proline in β_2_m has been shown to tip the energy landscape to favour aggregation over folding [83]. Detection and characterisation of rarely populated, partially unfolded states within the native ensemble under physiological conditions, therefore, will be crucial for our understanding of the mechanisms that link folding and aggregation energy landscapes.

## The aggregation side of the protein energy landscape

### Energy landscape roughness

Although the formulation of folding funnels provides a useful description of unimolecular folding in dilute solution, the behaviour of the polypeptide chain in the living cell, where the collision between molecules has to be considered, renders even more complexity onto the energy landscape view of protein conformational space [9,84,85]. Here, the competition between intramolecular and intermolecular interactions needs to be considered, resulting in a dramatic increase in landscape ruggedness. The schematic depicted in Fig. 1 attempts to depict the true complexity of the protein folding and aggregation energy landscapes, by including the wide range of different conformational states and the multitude of pathways available to each polypeptide chain as it circumnavigates the landscape [9]. In the living cell the relative depth of each energy well is not only determined by the polypeptide sequence, the temperature and the solution conditions as is the case for the folding of monomeric proteins, but is also governed by the protein concentration (Fig. 1b). Relative to the in-depth knowledge of the folding landscapes of simple, single domain monomeric proteins, relatively little is understood currently about the conformational states accessible to polypeptide oligomers. Energy minima on the aggregation side of the energy landscape might be poorly defined, as expected for broad ensembles of oligomeric states of similar energy that are rapidly interconverting, but could also be highly defined, as might be expected for higher order species such as protofibrils or fibrils. For example, the energy minimum of mature amyloid fibrils might be deeper and sharper than those of native monomeric proteins, as suggested by the rigidity of the amyloid fold, as well as the nucleation-dependent polymerisation mechanism [86]. Here, however, even under the same solution conditions a multitude of fibril morphologies can be formed simultaneously, highlighting the complexity and multiplicity of aggregation pathways [87,88]. As found for conformational variants of native, functional proteins, the conformational polymorphism common to many amyloid fibrils can also effect their biological properties [89].

The complex interplay between precursor structure, amyloid fibril morphology and infectivity has been described in depth by various groups for yeast prions [90]. The prion strain phenomenon, i.e. prion particles composed of the same polypeptide sequence that have distinct physiological effects, has recently been associated with conformation-dependent differences in prion structure and growth rates [91,92]. For example, different polymorphic, self-propagating forms of the same Sup35 sequence have been linked to variations in the strain phenotype, based on the growth rate and fibril stability (‘brittleness’) of amyloid fibrils [93]. The propagation of ‘conformational memory’ during templated fibril formation has also been shown to be the cause of species barriers [94]. Furthermore, solid-state NMR (ssNMR) experiments on Aβ_1−40_ amyloid fibrils, formed either quiescent or with agitation, have shown the propagation of conformational differences on an atomic level in seeding experiments [95]. The cause of these subtle, but highly significant, conformational changes is clearly situated in the assembly precursor states further upstream in the aggregation landscape [9]. Detailed characterisation of these species, therefore, will be crucial for developing a biophysical understanding of these phenomena. However, species populated early in the assembly reaction are rarely and only transiently populated, and may be extraordinarily heterogeneous, making their structural characterisation a difficult challenge for the years ahead.

### Amyloid fibril formation and protein aggregation diseases

An increasing number of human diseases has been linked to protein aggregation and the aberrant accumulation of protein deposits in different tissues and organs [96,97]. These pathological conditions include Alzheimer’s and Parkinson’s diseases, type II diabetes and the spongiform encephalopathies that are characterised by amyloid formation, as well as cystic fibrosis, α1-antitrypsin deficiency and amyotrophic lateral sclerosis (ALS) that involve less ordered aggregation. Together, these disorders are collectively known as protein misfolding diseases [98]. One of the most common groups of misfolding diseases are the amyloidoses, which involve the aggregation of specific proteins into ordered, insoluble, extra-cellular deposits [99]. These protein deposits contain fibrillar protein assemblies characterised by their specific dye-binding properties, cross-β X-ray fibre diffraction pattern and macroscopic long, straight and unbranched morphology [100]. Perhaps most importantly in the context of the energy landscape view of the protein universe, the observation that virtually any protein sequence can form amyloid fibrils given the appropriate solution conditions, led to the suggestion that the amyloid fold is the universal global free-energy minimum of all polypeptide chains that may assembly by generic mechanisms governed by the physicochemical properties of the polypeptide chain [101]. The amyloid fold, consisting of continuous β-sheets with β-strands oriented perpendicular to the fibril long axis, is remarkable in its commonality, stability and insolubility [100] and, as well as posing a threat to human health, offers exciting opportunities for exploitation in nano-biotechnology [102].

It is now becoming evident that Nature has also utilised the rigid-repeating structure of amyloid as productive structural or genetic components in some organisms [103]. Extraordinary conformational diversity is embodied by prions (including the mammalian prion, PrP, and the yeast/fungal prions Sup35, Ure2 and HET-s), which can assemble into protein aggregates with functionally distinct conformations, of which at least one is self-replicating [89]. This self-replication of conformational information enables prions to act as genetic elements with the ability to transmit diseases [104], to encode heritable phenotype traits [105], or to encrypt molecular memories [106]. Kelly and colleagues recently described the first known functional human amyloid, Pmel17, which templates melanin formation in melanocytes [107]. This finding demonstrates that amyloid is a fundamental, and at least in some cases non-pathological, protein fold, utilised by organisms from bacteria to humans. Although some common properties that link a specific polypeptide sequence to its aggregation behaviour are emerging (see below), the molecular mechanisms involved in differentiating between specific association into functional, non-pathogenic amyloid fibrils on the one hand, and the development of clinical symptoms and disease progression upon uncontrolled protein aggregation on the other, remain poorly understood and represent major future challenges.

## The mechanisms of protein aggregation

### Initiating amyloid fibril formation *in vitro*

**Fig. 3:**
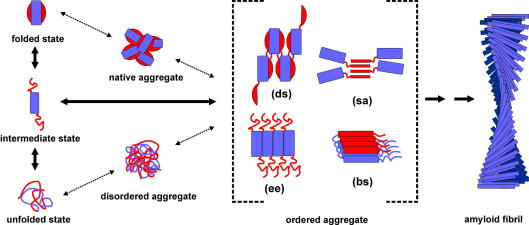
Proposed mechanisms of protein aggregation. Amyloid fibril formation for many proteins proceeds from intermediate partially folded states that are formed *via* partial unfolding of the native structure or *via* the partial structuring of unfolded polypeptides. Ordered aggregates associate *via* mechanisms such as domain swapping (ds), strand association (sa), edge-edge-association (ee) or β-strand stacking (bs). Self-association of these early oligomeric species, possibly involving further conformational changes, then leads to the formation of amyloid fibrils. The generic principles that govern this self-association process and the structure of the final amyloid fibril may depend critically on the polypeptide sequence and the solution conditions. In some proteins association of native-like monomers or non-specific self-association into disordered aggregates has been observed as the initial step in amyloid assembly, in the latter route the polypeptide adopts an ordered β-sheet structure within the initially disordered aggregate before amyloid fibril formation proceeds.

The generic nature of the aggregation process has enabled extensive studies of the transition between soluble precursor states and insoluble amyloid fibrils *in vitro* [108]. Here, the native conformation of globular proteins usually must be destabilised (e.g. by the addition of denaturants, low pH, high temperature, truncations or mutations) to allow protein aggregation to occur on a biochemically feasible timescale [109–111]. Specific protein destabilisation results in an increased population of partially folded conformations, whose exposed aggregation-prone regions enhance the probability of intermolecular interactions (Fig. 3). Which factors cause destabilisation of the native structure and the increase in the steady-state concentration of partially folded conformers *in vitro* and *in vivo* is now becoming clear for at least some proteins involved in amyloid disorders [108]. In the case of lysozyme, the aggregation of which is involved in hereditary systemic amyloidosis, single point mutations in the lysozyme gene are associated with fibril deposition in several tissues [112]. Two amyloidogenic variants (I56T and D67H) have been studied in detail and shown to be significantly less stable than the wild-type protein and, importantly, to lack the cooperativity of the native structure, leading to an increased concentration of partially folded states at equilibrium [82,113]. The same principle, i.e. enhanced amyloid propensity due to an increase in the concentration of a key amyloid precursor has been shown for transthyretin (TTR), a protein involved in familial amyloidotic neuropathy. In this case, amyloidogenic TTR variants have been shown to have a decreased tetramer stability and an increase in the tetramer dissociation rate constant, that together lead to an increase in amyloidogenesis [114]. However, for other proteins the full-length wild-type protein is the aggregating sequence and, for this set of proteins in particular, changes in the local environment or alterations of protein concentration are crucial for the onset of amyloidosis. For example in the case of β_2_m, the deposition of which results in dialysis-related amyloidosis [115], a rare partially folded conformation that is populated within the native ensemble has been identified as a key aggregation precursor [83]. The dissociation of β_2_m from the stabilising MHC class-I complex, together with the impairment of renal clearance (the normal site of β_2_m catabolism), leads to an up to 60-fold increase in the concentration of this intermediate species, subsequently resulting in the onset of aggregation and the deposition of the protein in amyloid plaques. Partially structured folding intermediates are believed to play a key role in fibril formation by many amyloidogenic proteins [83,116–118]. However, other assembly mechanisms have been suggested, including aggregation from a fully denatured state [119] or from the native state [120] (Fig. 3). A detailed analysis of conformational states populated in solution using powerful experiments able to detect and analyse the most rarely populated conformations (Table 1), therefore, is crucial for our understanding of the molecular events involved in the initiation of the amyloid cascade.

### Intermolecular association pathways

**Fig. 4:**
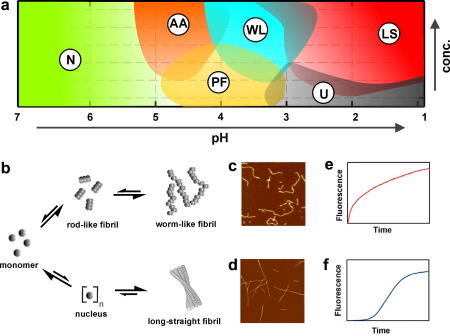
Pathway complexity of β_2_m amyloid fibril formation. (a) Schematic state diagram representing the different thermodynamic ground states observed upon incubation of β_2_m under different conditions. While the native protein (N) remains monomeric even at high protein concentration, acidification results in the protein unfolding to form partially folded (PF) or more highly unfolded forms (U). Above a critical concentration these species self-associate, forming amyloid fibrils with distinct morphological properties. Close to the protein’s pI, amorphous aggregates (AA) are formed, while worm-like fibrils (WL) and classic long-straight fibrils (LS) are formed at lower pH values. (b) Proposed model for competing pathways that lead to the formation of worm-like fibrils or long-straight amyloid fibrils. (c,d) AFM images of worm-like (c) and long-straight fibrils (d). All images are 1 μm^2^. (e,f) Worm-like fibrils form with nucleation-independent kinetics (e), whilst the formation of long-straight fibrils is nucleation-dependent and shows a clear lag-phase (f). Figure adapted from [126] with permission.

Several models for amyloid fibril formation have been suggested based on monitoring fibrillogenesis *via* microscopy, spectroscopic techniques and/or the binding of amyloid-specific dyes [121–124]. Classically, amyloid fibrils are formed in a nucleation-dependent manner, in which the protein monomer is converted into a fibrillar structure *via* a transiently populated aggregation nucleus [125]. After the rate-limiting step of nucleus formation aggregate growth proceeds rapidly by further addition of monomers or other assembly-competent species. Whereas the formation of the nucleus is thermodynamically unfavourable, its subsequent elongation is highly favourable and proceeds rapidly to the ultimate fibril structure. By contrast, the assembly of spherical oligomers and other prefibrillar forms occurs with nucleation-independent kinetics, and results in the formation of spherical particles or worm-like fibrils [122,126,127]. Here, polymerisation proceeds in the absence of a lag-phase. In some cases, oligomeric species have been suggested to be direct precursors of long-straight amyloid fibrils, whilst in other cases, an off-pathway role has been proposed [126,128–130]. In the case of β_2_m amyloid fibril formation, this question has be extensively studied using atomic force microscopy (AFM), mass spectrometry and analytical ultracentrifugation [126,127]. A schematic state diagram of the β_2_m aggregation landscape is illustrated in Fig. 4. While the incubation of native β_2_m at pH 7 does not result in fibril formation in the absence of fibrillar seeds, incubating β_2_m at pH values close to the pI results in the formation of amorphous aggregates, whilst at low pH values worm-like fibrils (around pH 3.6) or long-straight fibrils (around pH 2.5) are formed [88,111]. These states represent thermodynamic ground states on the energy landscape, whose population can be shifted by factors such as agitation, ionic strength, protein sequence or protein concentration [126]. Most importantly, assembly into worm-like fibrils has been shown to be off-pathway to the formation of long-straight fibrils, representing a competing reaction pathway (Fig. 4b). Using non-covalent mass spectrometry, a continuum of oligomeric species has been observed during the nucleation-independent formation of worm-like fibrils, while no oligomers larger then tetramers are detected during the assembly of long-straight fibrils [127]. Assembly of β_2_m into amyloid-like fibrils at low pH, therefore, involves significant conformational rearrangement from the initially highly dynamic, unfolded monomer at very low pH [32] to the compact, stable cross-β structure of amyloid. By contrast, formation of worm-like fibrils occurs from a partially folded conformer that retains significant native-like structure involving several of the native β-strands [131]. How the molecular characteristics of the monomeric precursor state and different kinetic routes influence the assembly reaction and the ultimate fibril morphology are currently unknown. However, the conceptual framework of the aggregation *versus* folding landscapes and the associated state diagrams of the assembly reaction (Fig. 4) provide a powerful framework on which to derive such insights and to design small molecules capable of inhibiting oligomer formation.

### Structural insights into oligomer and protofibril formation

Recent *in vitro* studies using electron microscopy (EM) and AFM have identified and characterised several intermediate structures populated during fibril formation, including small oligomers, membrane embedded pores, and protofibrils, the latter having a characteristic ‘beaded’ appearance [132,133]. Whether these structures form on-pathway, or are off-pathway to fibril formation, and which of these structures are actually the toxic ones are probably the most debated questions in this field today [134,135]. In the past few years, it has become evident that mature fibrils are biologically relatively inert species and that prefibrillar species, in general, are the most cytotoxic species on the energy landscape [136,137], although examples of toxicity associated with mature fibrils have also been reported [138]. An exciting study by Stefani and coworkers first showed the ‘inherent toxicity’ of early aggregates using protofibrils of the HypF protein and an SH3 domain, proteins not associated with any amyloid disease, whilst the mature amyloid fibrils formed from these proteins lack toxicity, reinforcing the view that fibrillar inclusions may serve a protective role [139]. While oligomeric species have been observed during fibril formation *in vitro* for many proteins, small oligomeric species have been isolated *in vivo* only rarely, examples including Aβ oligomers ranging from trimers to 56mers [128,132,140], which have been suggested to be involved in different aspects of the disease pathogenesis [141].

As soluble spherical oligomers are frequently observed during fibril formation, obtaining insights into their structural characteristics represents an important challenge. However, by contrast with mature fibrils, little is known about the molecular architecture of oligomers and protofibrils, based on their metastable nature and diverse morphologies [134]. Recent studies using hydrogen exchange and proteolysis have indicated a less-ordered structure for protofilaments compared with mature amyloid fibrils [142,143], in agreement with their lower β-sheet content suggested from FTIR and Thioflavin T (ThT) binding studies [144]. Using a plethora of spectroscopic techniques, Fink and coworkers were able to characterise the transient oligomers populated during assembly of the natively-unfolded protein, α-synuclein [144,145]. Here, early oligomers were shown to have significant β-sheet structure, exposed hydrophobic patches and a compact, partially folded structure. Determining the structure of oligomeric particles in atomistic detail remains a challenge for the future, requiring techniques able to decipher molecular architectures within ensembles of interconverting and commonly heterogeneous structures.

How monomers stack within amyloid-like structures has also been widely debated and, again, several models have been proposed (Fig. 3). By contrast with fibril formation from unfolded polypeptides, where substantial refolding has to occur (‘*refolding model*’), amyloid fibril formation from very native-like precursor states has been proposed to be achieved by a very limited set of conformational changes (‘*gain-of-interaction models*’), exposing a previously inaccessible surface to aid polymerisation [146]. Recent experimental evidence suggests that amorphous aggregated material may initiate amyloid fibril formation from unfolded polypeptides, with conformational rearrangements occurring subsequently to form ordered aggregate amyloid precursor states (Fig. 3) [147]. On the other hand, Chiti and coworkers have reported native-like prefibrillar assemblies in the aggregation pathway of AcP [120]. A direct stacking of native monomeric subunits has been proposed for models of TTR amyloid fibrils [148]. Recently, a generic cross-β spine model was proposed by Eisenberg and coworkers [149]. Here, the fibrils grow by providing a short polypeptide segment that stacks together to form the stable fibril spine. The recently determined high-resolution structures of the cross-β spines formed by 13 different short synthetic peptides based on X-ray diffraction of microcrystals, may envision the crystal-like packing of such a core structure, whilst at the same time revealing the structural diversity possible within the generic cross-β architecture of amyloid [150]. 3D domain swapping is a third mechanism by which monomers may stack within amyloid fibrils and their precursors. Here, homodimers or higher-molecular weight oligomers are formed by exchanging a specific domain, or part of a domain, between the assembling subunits [151]. The amyloid-like fibrils formed by RNase A have been shown to form by domain swapping in a ‘runaway’ manner [152] while retaining their enzymatic activity in the amyloid fold [153]. Although no single model is likely to account adequately for the properties of all amyloid fibrils formed under different conditions and from different polypeptide sequences, the models described to date may guide future experiments to determine whether there is indeed a generic route to the formation of a common amyloid fold.

### The amyloid fibril structure

Delineating the structure, at high resolution, of an amyloid fibril for any protein is still at the edge of our capabilities, despite the fact that the first definition of amyloid as possessing a generic cross-β structure was made nearly 40 years ago [154]. It has been difficult to obtain high-resolution structures of amyloid fibrils as these species are insoluble and non-crystalline. However, recent advances in experimental methods are starting to provide a detailed picture of the amyloid architecture [100,155]. Amyloid is defined in terms of empirical observations from X-ray fibre diffraction, EM and specific chemical staining [99,156,157]. The cross-β fibre diffraction pattern has two characteristic signals, a meridional reflection at 4.7 Å along the fibril axis and a more diffuse equatorial reflection around 10 Å perpendicular to the fibre direction, representing the continuous β-sheet and the inter-sheet packing, respectively [158]. Variation in the models of the continuous cross-β structure, such as β-helical [159,160] or nano-tube architectures [161], have also been suggested and are still under debate [162]. EM and AFM studies have shown that amyloid fibrils are straight, unbranched, and about 70–120 Å in diameter [163]. Furthermore, birefringence under cross polarisers upon staining with Congo red, and a fluorescence shift after staining with ThT, are also classical features of the amyloid fold [157]. Although these characteristics have been tested for *ex vivo* amyloid [164], detailed information about the structure of amyloid is usually gathered from amyloid-like fibrils grown *in vitro*, since these lack the extraneous factors commonly associated with amyloid *ex vivo* [165] and careful control of the growth conditions *in vitro* can lead to much more homogeneous fibril preparations.

**Fig. 5:**
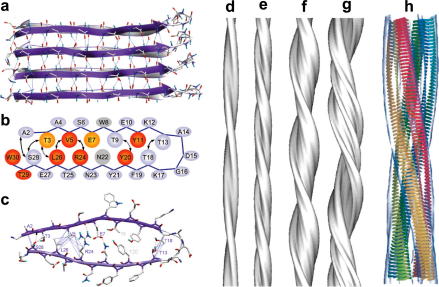
General structural motifs of amyloid-like fibrils. (a–c) Structural model for protofilaments formed *in vitro* by a WW domain. (a) Perpendicular view to the fibril axis, indicating the continuous β-sheet hydrogen bond structure (dotted lines). (b) Cartoon representation of the non-native β-strand-loop-β-strand motif adopted in the amyloid structure. Structural restraints from ssNMR measurements are indicated by arrows and side chains eliminating (red) or reducing (orange) amyloid fibril formation when mutated to alanine are highlighted. (c) Atomistic representation of the tight packing between strands (viewed along the fibril axis as in (b)). (d–g) Surface representation of 3D maps obtained using cryoEM for insulin fibril structures. The fibrils contain either two (d), four (e) or six (f and g) protofilaments. (h) Proposed β-strand model for insulin fibrils containing four protofilaments. Figure adapted from references [119] and [87] with permission.

Recent advances in cryoEM, ssNMR, electron paramagnetic resonance (EPR) and X-ray crystallography are now beginning to provide valuable, detailed, information on the amyloid fibril architecture. Using ssNMR, Ferguson and coworkers have obtained the structure of the amyloid fibrils formed from a CA150-WW domain (Fig. 5a–c) [119]. In this structure, each molecule forms a pair of β-strands stacked *via* specific side chain interactions, a model that has also been proposed for the amyloid fold of the Aβ_1–40_ peptide [166], as well as a peptide fragment from β_2_m [167]. The β-strands are packed in a parallel, in-register manner along the fibril long axis (Fig. 5a). A similar, double β-strand-turn-β-strand motif was recently proposed for the amyloid-like fibrils of HET-s (218–289), a prion protein domain from filamentous fungi [168]. Experimental techniques such as hydrogen exchange, limited proteolysis, fluorescence and proline-scanning mutagenesis [143,169–172] are important in deriving models for the fibril architecture, providing information including the fraction of the polypeptide chain comprising the amyloid fibril core, as well as the specific packing of the amino acid side chains. While these experiments provide information about local structural order, the overall fibril morphology, including the packing of protofilaments, is best described using AFM or cryoEM. Using the latter technique, in combination with single particle analysis and helical reconstruction, Saibil and coworkers were able to describe the 3D structures of amyloid fibrils from insulin, SH3 domains and PrP, including detailed information about the arrangement of their protofilaments [87,173,174]. In the example of insulin (Fig. 5d–g), different numbers of protofilaments, composed of relatively flat β-sheets with a left-handed twist (Fig. 5h), were shown to account for four different fibril morphologies [87]. Therefore, the α-helical structure of native insulin must reorganise substantially to form the amyloid cross-β fold. Determining how these structures relate to those of amyloid fibrils formed from more complex protein structures, as well as those associated with *in vivo* disease, remain significant future challenges.

## Determinants of protein aggregation

### Theoretical concepts and computational models

**Fig. 6:**
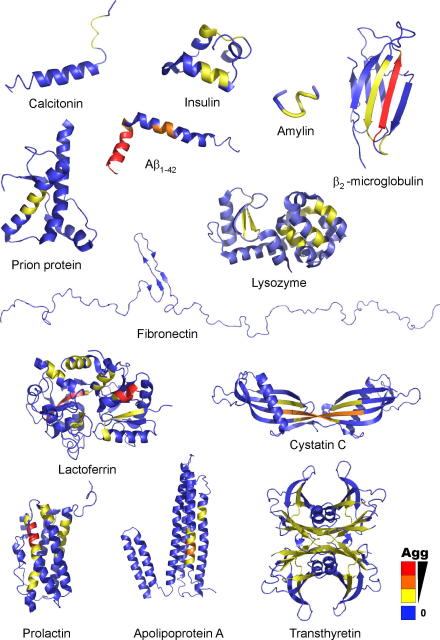
Representative structures of proteins involved in disease-related amyloid fibril formation. The polypeptides are coloured according to the aggregation tendency of their amino acid sequences predicted using the algorithm TANGO [187]. Sequences shown in blue are predicted to have no β-aggregation propensity, while polypeptide stretches coloured in yellow, orange and red indicate an increasing propensity to aggregate. Notably, the peptide structures were obtained in the presence of fluoroalcohols (calcitonin and Aβ_1–42_) or SDS micelles (amylin), and these sequences might be substantially less ordered in the absence of these additives. Note also that for insulin, amylin and calcitonin, the pro-peptides as well as the mature sequences have been implicated as potentially amyloidogenic [225–227].

The extreme diversity of proteins associated with amyloid fibrils in human disease is illustrated in Fig. 6. These structures range from natively unfolded polypeptides, through polypeptides possessing extensive α-helical structure, to proteins containing β-sheet structure exclusively or in part. In addition, monomeric proteins, small peptides and proteins found naturally as multimers under physiological conditions, are all known to cause human amyloid disease (Fig. 6). The ordered aggregation of globular proteins has been shown to require the partial unfolding of the native state [108], such that a precursor state is populated that exposes aggregation-competent regions that are usually protected against forming intermolecular interactions in the native protein. Although the high-resolution structural characterisation of amyloid precursor species has been hindered to date because of their conformational flexibility, rarity and inherent heterogeneity, computer simulations and theoretical studies are now beginning to shed light on the key properties of these aggregation precursor species, as well as the molecular events in the early stages of the assembly process, and the stability of the final amyloid fold [175,176]. Importantly, these studies have revealed that the properties inherent in the sequence of the polypeptide chain play a crucial role in governing aggregation [85]. In simulations of polypeptide self-assembly the polypeptide chains were shown to undergo a hydrophobic collapse into partially ordered or amorphous aggregates, followed by the transition to ordered β-sheet aggregates by optimising intermolecular interactions [177,178]. Different subunit alignments have been observed, involving parallel or antiparallel structures and in- or out-of-register β-strands, dependent on the amino acid sequence and its length [179,180].

The residues key to the aggregation process are thought to be different from those important in driving correct folding of the polypeptide chain [181], although the major driving forces (the formation of hydrogen bonds and the burial of hydrophobic surface area) are commonly and critically involved in both processes. Although a large part of the polypeptide chain may be involved in the fibril structure [182], it is clear that some amino acid sequences are more prone to aggregation than others [183]. In fact, recent evidence supports the idea that short stretches of amino acids can trigger the aggregation of larger, normally soluble proteins [184]. Thus, akin to a protein folding reaction where only a few residues define the critical folding nucleus, but many residues are required to support the structure of the folding transition state [37], key residues may also be important in governing the assembly of the polypeptide chain into amyloid fibrils. From a systematic analysis of more than 50 mutational variants of AcP, Chiti et al. rationalised the propensities of some sequences to aggregate more rapidly than others, based simply on the physicochemical characteristics of the polypeptide chain: hydrophobicity, secondary structure propensity and charge [185]. Combining these three parameters with further factors (e.g. peptide concentration, pH and ionic strength of the solution) not only allows the prediction of aggregation propensity, but also allows the prediction of aggregation rates [186] and, therefore, provides an important step towards a quantitative understanding of factors influencing protein aggregation. Based on similar principles, Serrano and coworkers have developed a generic algorithm, TANGO, which predicts the propensity of a given, linear amino acid sequence to form β-sheet aggregates, [187]. Although amyloid fibril formation propensity is not specifically predicted, the result of this algorithm is illustrated in Fig. 6 for proteins known to aggregate into amyloid fibrils associated with human disease. Notably, aggregation-prone stretches can be found in α-helical regions, as well as regions forming β-sheet structures in the native protein. In addition, regions that are either exposed or buried in the native fold are predicted to be involved in amyloid formation, reiterating the need for partial unfolding of the native protein during the conversion to the β-sheet structure of amyloid. Importantly, the general success of these algorithms [85,176] in predicting aggregation-prone regions in intact proteins indicates that short stretches of the sequence might drive the aggregation of the entire polypeptide chain and allows rationalisation of protein aggregation behaviour on the basis of side-chain composition within these specific regions. Further developments of these methods to include the effect of residual structure in unfolded states, as well as structure in partially folded amyloidogenic intermediates are now needed to quantitatively understand and predict protein aggregation processes *in vitro*. Building on these successes it may also be possible to envisage in the future, the use of such methods to predict aggregation propensity *in vivo*, of immense potential importance for the design of prospective therapies.

### Intrinsic gatekeepers

**Fig. 7:**
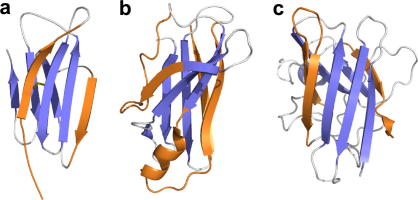
Schematic illustration of the importance of edge-strands in the aggregation of β-sheet proteins. Local unfolding of the protective edge-strands (orange) has been implicated in amyloid fibril formation for (a) β_2_m using NMR spectroscopy [83], (b) TTR using hydrogen exchange experiments [192] and (c) SOD using protein engineering [193].

Using the power of aggregation prediction algorithms, the aggregation propensity of full proteomes has been analysed in detail [188,189]. From this study a clear evolutionary pressure was apparent, in which the aggregation propensity of the sequences of functional proteins was found to be reduced significantly compared with random polypeptide sequences. Although aggregation usually cannot be completely suppressed because of the functional and structural constraints of the native fold, aggregation is reduced substantially by placing charged residues and/or β-sheet breaking residues adjacent to aggregation-prone segments [189]. Proteins thus appear to have evolved determinants to prevent aggregation, while maintaining the ability to fold. For example, proline residues frequently found in membrane α-helices are thought to maximise correct folding by preventing misfolded (β-sheet) conformations [190]. In addition, the edge-strands of native β-sheet proteins are protected from forming intermolecular hydrogen bonds by a number of positive design features, such as β-bulge structures or charged residues, that protect exposed edge-strands from improper intermolecular interactions [191]. The importance of edge-strands in the aggregation of β-sheet proteins under native conditions has recently been observed experimentally. A folding intermediate, populated in the native state ensemble of β_2_m under physiological conditions, has been identified as the species involved in amyloid fibril formation and has been characterised by NMR [83]. While retaining a native-like structure in five of the seven β-strands of the native immunoglobulin fold, the edge-strands A and D that cap the central core show large structural deviations from the native conformation (Fig. 7a), strongly suggesting that loss of these protective features triggers aggregation. Further evidence comes from studies on TTR [192] and SOD [193], where hydrogen exchange and protein engineering, respectively, have indicated that the edge-strands are the most labile strands of these β-sheet proteins and that specific local unfolding of these regions might initiate intermolecular interactions.

It has been suggested that the high conservation of proline residues in the fibronectin type III superfamily can be rationalised on the grounds that these residues prevent aggregation by interfering with the formation of β-sheet structure [194]. A similar ‘gatekeeper’ role has recently been proposed for glycine residues [195]. Next to being utilised as conformational switches [196], proline residues may also provide a protective mechanism, by introducing high energy barriers on the folding energy landscape that disfavour the unfolding of the native state and the formation of aggregation-prone partially folded species [83]. In the case of β_2_m, the isomerisation of only a single, conserved proline residue (Pro32) from the native *cis*-conformation into the *trans*-form switches the native protein into an aggregation-prone state. As a result of a high-unfolding barrier, this intermediate is only marginally populated in the native state ensemble, and addition of copper ions or an increase in protein concentration can shift this equilibrium towards aggregated states [83,197]. Interestingly, a well-defined ratio between hydrophobic and polar residues has been found in all soluble native proteins, with the outcome that long stretches of alternating polar and non-polar amino acids, in general, is avoided in native polypeptide sequences [198]. Intrinsically unfolded proteins seem to be a good model to discover strategies for avoiding aggregation, as they are less aggregation prone compared with compact native proteins [199]. Indeed, the amino acid propensity for being intrinsically unfolded and the propensity for aggregation are anti-correlated [85]. Interestingly, specific sequence variations have also been discovered in multidomain proteins [200], such as the muscle protein titin. Here, neighboring subunits show less than 40% sequence identity, experimentally shown to be the threshold of aggregation compatibility [201], highlighting the specificity of protein aggregation [202–204]. In addition to the pressure to fold rapidly to the native state, the suppression of off-pathway aggregation reactions has been precisely encoded by evolution in the development of today’s protein sequences.

### Cellular protein quality control

Next to the intrinsic features of the polypeptide chain that govern the partitioning between folding and aggregation, a battery of cellular components forms the cell’s quality-control machinery and ensures the correct folding of proteins or the rapid degradation of mutated or misfolded polypeptides. The folding of newly synthesised proteins to their native conformations involves the sequential action of multiple molecular chaperones [205]. Molecular chaperones, most of which are stress inducible as heat shock proteins (Hsp), act in a tightly controlled ATP-dependent manner to bind and release unfolded or misfolded polypeptides [206]. Chaperones (including the Hsp100, Hsp90, Hsp70 and Hsp60 families, for example) prevent aggregation by smoothing the energy landscape and, therefore, decreasing the population of partially folded species [207]. This is achieved by enhancing the rate of folding, protecting aggregation-prone intermediates from intermolecular interactions, or targeting misfolded proteins to the degradation machinery [208]. Muchowski and coworkers were able to show that the cellular chaperones Hsp70 and Hsp40 attenuate the formation of spherical and annular oligomers, whilst favouring formation of fibrillar species [209], rationalising the finding that these chaperones also suppress neurodegeneration in animal models for Huntington`s and Parkinson’s diseases [210]. In addition to the Hsp70 system, a study by Behrends et al. showed that the cytosolic chaperone TRiC interferes with the aggregation of polyglutamine proteins, driving the assembly reaction into soluble 500–600 kDa oligomers without detectable cytotoxicity [211]. Even through chaperones like Hsp104 can resolubilise microaggregates [212], and have been shown to be essential for prion propagation in yeast [213], the mechanisms for the solubilisation and degradation of large proteinaceous deposits are currently poorly understood.

Cells use two major protein degradation systems for the quality control of newly synthesised proteins and the disposal of old or damaged proteins. As much as 30% of the newly synthesised protein pool is degraded due to ineffective folding [214] *via* the ubiquitin–proteasome system that removes individual proteins that have been marked for degradation [215]. The other system, autophagy, relies on vesicles to engulf portions of the cytoplasm and deliver them to lysosomes. This mechanism is used to turn over long-lived proteins and organelles [216]. A highly activated autophagic response is observed in Huntington’s, Parkinson’s and Alzheimer’s diseases, presumably as a protective response to the accumulation of toxic proteins or aggregates [217]. In addition, two recent reports used autophagy-gene knock-out mice to show that the lack of autophagy is associated with progressive neurodegeneration, leading to the accumulation of ubiquitin-containing inclusion bodies, independent of the proteasome system [218,219]. However, even for proteins that fold successfully to their native structure and hence escape the cellular quality control machinery, random conformational fluctuations of the native protein can lead to the transient formation of aggregation-prone intermediate states (see above). In the crowded environment of the cell such species may have an increased propensity to aggregate, forming small oligomers or larger particles that initiate the amyloid cascade. Especially in age-related amyloidosis this may lead to the accumulation of large quantities of partially folded proteins and the saturation of the capacity of the quality control machinery, exacerbating the formation of intracellular aggregates before refolding or degradation is possible [220]. These examples clearly suggest that the necessity to avoid aggregation has experienced a similar evolutionary driving force as the pressure to fold successfully into a unique three-dimensional structure.

### Amyloid therapies

Since the identity of the toxic species for many amyloid diseases currently remain unknown, and their structures remain elusive, approaches for the prevention of toxicity in amyloidosis are still in their infancy [221]. However, attractive therapeutic approaches are based on the idea of smoothing the protein landscape to prevent accumulation of aggregation-prone or toxic species. *In vitro* studies of TTR, for example, have shown that small molecules (‘chemical chaperones’) that mimic the binding of natural ligands and stabilise the native tetrameric structure are effective anti-amyloid agents. Dobson and coworkers used a single-domain fragment of a camelid antibody to rescue the amyloidogenic lysozyme variant D67H from amyloid fibril formation [222]. Interestingly, this was achieved by increasing protein stability and restoring the cooperativity between the two structural domains in the native protein, reducing the number of global unfolding events and decreasing the probability of forming partially unfolded states [222]. While the properties of native proteins are encoded by the amino acid sequence, amyloid deposition pathways *in vivo* also depend strongly on a number of cofactors including serum amyloid P (SAP) component, apolipoprotein E and glycosaminoglycans, that generically bind and stabilise the fibrillar state [165]. In the absence of these factors, fibrils can be depolymerised, offering other routes for therapeutic intervention [223,224]. A clear understanding of the mechanism of the association of these cofactors with amyloid fibrils may expose further possibilities of reversing amyloid deposition, presuming that this does not result in an increase in the production of toxic species. Evolution has successfully managed to avoid amyloid fibril formation to an astonishing degree, considering the vast amount of polypeptide sequences crowded *in vivo*. However, the advances in medical care that are allowing life to an increasing age are resulting in a marked increase in protein misfolding diseases in our increasingly aged population. Detailed analysis of factors (intrinsic as well as environmental) that alter the balance between protein folding and aggregation, therefore, will be essential for developing future medical interventions against these debilitating diseases.

## Summary and future perspectives

Research over the last decade has provided valuable insights into the biophysical principles of protein misfolding and aggregation and their relationship to human disease. Generic principles have emerged from these studies such that detailed descriptions of both productive folding mechanisms and the aggregation-side of the energy landscape are beginning to emerge. Importantly, recent studies have allowed the protein folding and aggregation energy landscapes to be linked by defining the species that are common to both pathways. As outlined above, we are now able to rationalise the flux between protein folding and protein aggregation based in part on the intrinsic characteristics of a given polypeptide sequence, modulated by extrinsic factors such as the solution conditions, the binding of co-factors or cellular stress. Furthermore, methods are now beginning to emerge that allow detailed descriptions of different species on the folding and aggregation landscapes, as well as the pathways that connect them. Although generic concepts of protein misfolding and fibril formation have been established, the major challenge for future studies will be in the refinement and improvement of these models, so that the shape of the folding and aggregation landscapes can be predicted under conditions relevant to the living cell. Linking biophysical observations (e.g. the population of oligomers and their structural properties) with the progress of disease (e.g. neuronal dysfunction and cell-death) will be a further major task. Some of the most exciting questions for the future include: What is the structural mechanism of aggregation initiation? How is the stability and rigidity of amyloid fibrils structurally manifested? Is there a common mechanism of oligomer assembly and cytotoxicity? Can we rationally design therapies against these diseases? How has Nature utilised the otherwise toxic amyloid fold? We still have much to learn and there are likely to be many exciting surprises ahead as we try to achieve better understanding of the tight balance between protein folding and aggregation. Understanding the complex energy landscape and determining the structural properties of rare and heterogeneous species populated transiently at different stages of folding and aggregation will undoubtedly play a significant role in this future progress.

## Figures and Tables

**Table 1:** Examples of experimental approaches for characterising conformational ensembles populated on the protein energy landscape

Experiment	Technique	Species
*Kinetic*		
Folding/assembly	Spectroscopy (absorption, fluorescence, infra-red, circular dichroism etc.)	U, I, N, O, A
	NMR (real time, relaxation and line-shape analysis etc.)	U, I, N
	Mass spectrometry	U, I, N, O, A
	Single molecule experiments (FRET, FCS etc.)	U, I, N, A
	Protein engineering (phi-value analysis^∗^ etc.)	U, I, N
	Specific dye binding (ANS, Thioflavin T etc.)	U, I, N, O, A
	Hydrogen–deuterium exchange^∗^	U, I, N, O, A
	Turbidity and light-scattering	N, O, A
	Chemical cross-linking	O, A
*Equilibrium*		
Structure	X-ray crystallography	N
	Fibre diffraction	A
	Electron paramagnetic resonance (EPR)	A
	Solution NMR (NMR order parameters^∗^, residual dipolar couplings^∗^, nuclear Overhauser effects^∗^ etc.)	U, I, N
	Solid state NMR	O, A
	Cryo-electron microscopy	A
Conformation	Spectroscopy (see above)	U, I, N, O, A
	Electron and atomic force microscopy (EM and AFM)	O, A
	Analytical ultracentrifugation	U, I, N, O
	Gel permeation chromatography	U, I, N, O
	Calorimetry	U, I, N
Dynamics	NMR (relaxation measurements^∗^, residual dipolar couplings^∗^, spin-labelling techniques^∗^ etc.)	U, I, N
	Hydrogen–deuterium exchange^∗^	U, I, N, O, A
	Denaturant and proteolysis stability	U, I, N, O, A

The observable states are grouped into native state (N), intermediate state (I), unfolded state (U), oligomeric states (O) and amyloid fibrils (A). Experimental data from techniques marked by an asterisk might be used as restraints in molecular dynamics simulations. Adapted from [9].

## Footnotes

1 *Abbreviations used:* Aβ, amyloid-beta peptide; AcP, acylphosphatase; AFM, atomic force microscopy; β_2_m, β_2_-microglobulin; HX, hydrogen exchange; CI2, chymotrypsin inhibitor 2; CD, circular dichroism; EM, electron microscopy; EPR, electron paramagnetic resonance; FRET, Förster resonance energy transfer; Hsp, heat shock protein; HX, hydrogen exchange; Im7, immunity protein 7; MD, molecular dynamics; NMR, nuclear magnetic resonance; PUFs, partially unfolded forms; PrP, prion protein; ssNMR, solid state NMR; SOD, superoxide dismutase; ThT, Thioflavin T; TTR, transthyretin.
